# A familiar study on self-limited childhood epilepsy patients using hIPSC-derived neurons shows a bias towards immaturity at the morphological, electrophysiological and gene expression levels

**DOI:** 10.1186/s13287-021-02658-2

**Published:** 2021-11-25

**Authors:** Mariana L. Casalia, Juan Cruz Casabona, Corina García, Verónica Cavaliere Candedo, Héctor Ramiro Quintá, María Isabel Farías, Joaquín Gonzalez, Dolores Gonzalez Morón, Marta Córdoba, Damian Consalvo, Gustavo Mostoslavsky, Francisco J. Urbano, Juana Pasquini, Mario Gustavo Murer, Lorena Rela, Marcelo A. Kauffman, Fernando J. Pitossi

**Affiliations:** 1 Institute Leloir Foundation- IIBBA-CONICET, Buenos Aires, Argentina; 2 CONICET and Laboratorio de Medicina Experimental “Dr. J Toblli”, Hospital Alemán, Buenos Aires, Argentina; 3 Consultorio y Laboratorio de Neurogenética, Centro Universitario de Neurología “José María Ramos Mejía” Facultad de Medicina, UBA & Instituto de Investigaciones en Medicina Traslacional, Facultad de Ciencias Biomédicas, Universidad Austral-CONICET, Buenos Aires, Argentina; 4 Center For Regenerative Medicine (CReM) of Boston University and Boston Medical Center, Boston, USA; 5 Department of Physiology, Molecular and Cellular Biology “Dr. Héctor Maldonado”, Faculty of Exact and Natural Sciences, University of Buenos Aires, IFIBYNE-CONICET, Buenos Aires, Argentina; 6 Faculty of Pharmacy and Biochemistry, University of Buenos Aires, Buenos Aires, Argentina; 7 Universidad de Buenos Aires, Facultad de Medicina, Departamento de Ciencias Fisiológicas, Grupo de Neurociencia de Sistemas, Buenos Aires, Argentina; 8 Universidad de Buenos Aires - CONICET, Instituto de Fisiología y Biofísica Bernardo Houssay (IFIBIO), Buenos Aires, Argentina

## Abstract

**Background:**

Self-limited Childhood Epilepsies are the most prevalent epileptic syndrome in children. Its pathogenesis is unknown. In this disease, symptoms resolve spontaneously in approximately 50% of patients when maturity is reached, prompting to a maturation problem. The purpose of this study was to understand the molecular bases of this disease by generating and analyzing induced pluripotent stem cell-derived neurons from a family with 7 siblings, among whom 4 suffer from this disease.

**Methods:**

Two affected siblings and, as controls, a healthy sister and the unaffected mother of the family were studied. Using exome sequencing, a homozygous variant in the FYVE, RhoGEF and PH Domain Containing 6 gene was identified in the patients as a putative genetic factor that could contribute to the development of this familial disorder. After informed consent was signed, skin biopsies from the 4 individuals were collected, fibroblasts were derived and reprogrammed and neurons were generated and characterized by markers and electrophysiology. Morphological, electrophysiological and gene expression analyses were performed on these neurons.

**Results:**

Bona fide induced pluripotent stem cells and derived neurons could be generated in all cases. Overall, there were no major shifts in neuronal marker expression among patient and control-derived neurons. Compared to two familial controls, neurons from patients showed shorter axonal length, a dramatic reduction in synapsin-1 levels and cytoskeleton disorganization. In addition, neurons from patients developed a lower action potential threshold with time of in vitro differentiation and the amount of current needed to elicit an action potential (rheobase) was smaller in cells recorded from NE derived from patients at 12 weeks of differentiation when compared with shorter times in culture. These results indicate an increased excitability in patient cells that emerges with the time in culture. Finally, functional genomic analysis showed a biased towards immaturity in patient-derived neurons.

**Conclusions:**

We are reporting the first in vitro model of self-limited childhood epilepsy, providing the cellular bases for future in-depth studies to understand its pathogenesis. Our results show patient-specific neuronal features reflecting immaturity, in resonance with the course of the disease and previous imaging studies.

**Supplementary Information:**

The online version contains supplementary material available at 10.1186/s13287-021-02658-2.

## Background

Self-limited Childhood Epilepsies (SLCE) are common, age-restricted focal epilepsy syndromes that affect 20/100.000 new children every year [1]. Two occipital variants of SLCE are eponymously known as Gastaut and Panayiotopoulos types. However, the electro-clinical overlap shown by patients affected by occipital SLCE precludes the use of rigid classifications schemes [2]. Therefore, the more general term SLCE is favored for patients suffering from focal epilepsy during the first 2 decades of life that frequently remit during adulthood [3].

The existence of genetic determinants in the etiology of SLCE has long been proposed. Familial aggregation has repeatedly been described for SLCE and occasional reports of disease-causing mutations in occipital SLCE patients have been published. However, it is still unclear if monogenic forms of occipital SLCE exist at all or, if their genetic architecture is better explained by a complex inheritance model. Polygenic models of neurodevelopmental disorders such as SLCE recognize core genes belonging to pathways that play a central role in the disease, and peripheral genes spread across the genome that may have key effects on heritability through trans-regulatory effects on the core genes [4]. Emerging phenotypes, especially in the case of neurodevelopmental disorders such as some forms of epilepsy, depend not only on genetic variability but also on the stochastic execution of developmental programs [5]. Moreover, recently, atypical maturation of white matter microstructure has been found in benign epilepsy with centrotemporal spikes contributing to epileptogenesis and suggesting a maturation defect underlying this disease [6].

Despite its high impact in the study of neurological disorders, induced pluripotent stem cells (iPSC)-derived neurons have been scarcely used to investigate epilepsies [7, 8]. In particular, no in vitro model is available to study SLCE.

Here, we have studied a family of 7 siblings, among whom 4 suffer from occipital SLCE and show high intra-familial phenotypic variability. Since this pattern of disease suggests an underlying genetic component, using exome sequencing, a homozygous variant in the FYVE, RhoGEF and PH Domain Containing 6 gene (FGD6) was identified as a putative genetic factor that could contribute, under a possible oligogenic model, to the development of this familial disorder. We have generated hiPSC-derived neurons from two affected siblings compared to two familial controls that showed morphological and functional abnormalities that could clarify some of the mechanisms leading to SLCE. In addition, these lines could serve as cellular platforms for drug discovery in SLCE.

## Materials and methods

### Patient selection

We performed Exome Sequencing (ES) in 2 affected subjects from a family of 7 siblings following similar procedures to those described by us previously [9]. In brief, Blood genomic DNA was captured with the Agilent SureSelect Human All Exon Capture V2 Kit and sequenced as an outsourced service from a commercial provider (Macrogen Inc). Sequence processing, alignment (with a Burrows-Wheeler algorithm), and variant calling were performed according to the Broad Institute Genome Analysis Toolkit (GATK v.3) Best Practices, and variants were annotated with ANNOVAR using a custom pipeline. To identify potentially pathogenic variants, we selected variants under a recessive inheritance model affecting coding and splice sites that were present at minor allele frequencies (MAFs) lower than 0.01% in public databases (gnomAD). We classified selected variants following ACMG recommendations, discarding thereafter those flagged as benign or likely benign. We selected for segregation analysis by Sanger sequencing in the remaining family members those VUS variants (homozygous and compound heterozygote) that were present in both siblings studied by ES. From a list of 47 candidate genes, only the FGD6 variant showed perfect segregation in the 7 family members that were assessed.

### Fibroblast derivation

All the work was done under the approval of Ramos Mejía Hospital and Leloir Institute Foundation ethical committees. All individuals participating in the study and/or their authorized representative were provided and signed a written informed consent previously approved by the ethical board of the project leading institutions. A 3 mm diameter skin biopsy of each donor was obtained as starting material for the fibroblast derivation. Briefly, under sterile conditions the biopsy was rinsed twice in PBS supplemented with 100 U/ml of Penicillin (Invitrogen), 100 ug/ml Streptomycin (Invitrogen) y 0.3 ug/ml Amphotericin B (Invitrogen) accordingly to [10]. After the removal of fatty tissue the epidermis was placed facing down onto a plate pretreated with 0.1% gelatin (SIGMA) and fetal bovine serum (FBS, GIBCO) in fibroblast maintaining medium (DMEM high glucose, Invitrogen) supplemented with 20% of FBS (GIBCO), 2 mM Glutamine (SIGMA), 0.3 ug/ml Amphotericin B (Invitrogen), 100U/ml Penicillin (Invitrogen) and 100 ug/ml Streptomycin (Invitrogen), renewing every 5 days. After 20 days or reaching a 50% of confluence the cells were treated for 5 min at 37 °C with trypsin (Invitrogen). At this point the cells were characterized as fibroblast by immunofluorescence and morphology according to [10]. The remaining tissue was re-plated to further obtain fibroblasts and later cryopreserved for potential use.

### Cell reprogramming

100.000 fibroblast were transduced with the lentiviral vector STEMCCA [11] at a multiplicity of infection (MOI) of 1. After 72 h cells were placed in stem cell medium (DMEM/F12 (Invitrogen) supplemented with 20% Knockout Serum Replacement (Invitrogen), 0.1 mM non essentials amino acids (Invitrogen), 0.1 mM 2-Mercaptoethanol (Invitrogen), 2 mM Glutamine (SIGMA), 0.3 ug/ml Amphotericin B (Invitrogen), 100 U/ml Penicillin (Invitrogen) and 100 ug/ml Streptomycin (Invitrogen) supplemented with 20 ng/ml FGF2 (Peprotech). Media was changed every 48 h. After 7 days the cells were placed onto a feeder layer of irradiated inactive murine embryonic fibroblast (MEF). After 20 days, the colonies resembling embryonic stem cells [12] were mechanically selected and individualized for further characterization as induced pluripotent stem cells (hiPSC). 4–16 hIPS clones from each patient or control were obtained after reprogramming for further characterization as follows: patient 1:15 clones, patient 2:16 clones; control 1:8 clones, control 2:4 clones. 4 clones from each patient and controls have been extensively characterized during the quality controls to define each clone as bona fide hIPSC (as shown in Additional file 10: Fig. S2). Between 4 and 5 hiPSC clones were differentiated: patient 1:5 clones, patient 2:5 clones; control 1:5 clones, control 2:4 clones. For the characterization of NE, between 2 and 3 independent NE clones were analyzed for each patient or control samples.

### PCR and RT-PCR

To confirm the integration of the lentiviral vector in the reprogramed cells a PCR using primers designed to amplify regions specifically found in the STEMCCA, but not in the endogenous genes was used (Additional file 2: Table S2). To detect the endogenous expression of pluripotency genes, RT-PCR was performed according to [13, 14] (Additional file 3: Table S3). Briefly, conditions for the RT-PCR were 1 cycle at 95 °C for 2′, 4 cycles at 95 °C for 30″, 56 °C for 45″ and 72 °C for 1′, after 35 cycles at 95 °C for 30″, 58 °C for 45″, 72 °C for 1′ and the final extension round at 72 °C for 10. To detect lineage dependent genes in embryoid bodys (EB) a quantitative RT-PCR was performed. Conditions were 1 cycle at 95 °C for 2′, 39 cycles at 95 °C for 30″, 64 °C for 45″, 72 °C for 45″ (Additional file 4: Table S4).

### Karyotyping

For hIPS karyotyping, cultured colonies were splited into ~ 100 very small aggregates and plated onto 10 cm^2^ culture plates on irradiated mouse embryonic fibroblasts. The cultures were grown for 48 to 120 h, treated with 17.5 ul/ml of Karyomax (Gibco, 10 ug/ml), and subsequently trypsinized until single cells were left. Cells were collected and incubated with hypotonic solution (0.075 M KCl) during 30 min and then fixated with 3:1 methanol/glacial acetic acid. For Fibroblast karyotyping 100.000 cells were plated onto a 20 cm^2^ culture plate, grown for 48 h and then treated with Karyomax as before, during 2–4 h. The procedure was the same as with the hIPS cultures but cells were fixed for 12 min. The chromosome analysis was performed using a trypsin-Giemsa banding technique. At least 20 metaphases were analyzed for each sample.

### Immunofluorescence

hiPSC clones or EBs were fixed for 10’ with 4% PFA at room temperature. Briefly, cells were blocked with 1% donkey serum in TBS (Tris 0.1 M, ClNa 0.15 M) with 0.1% Triton X-100 for 1 h at room temperature. Samples were then incubated with the first antibody diluted in blocking solution at 4 °C (Additional file 4: Table S4). After 12 h the samples were washed 2 times with TBS in 0.1% Triton X-100, one time with 0.1 M PB (Na_2_HPO_4_, KH_2_PO_4_) for 10′. Then the cells were incubated during 2 h at room temperature with the secondary antibody in 0.1 M PB. Nucleuses were stained with DAPI (SIGMA). Images were obtained with an Olympus BX60 microscope. Neuroepithelial tissue (NE) was fixed with 4% PFA for 10 min at 4 °C, washed 3 times with 0.1 M PB and incubated O.N. at 4 °C in 30% sucrose. The next day the tissue was place in a cryopreservative solution (3 volumes of glycerol, 3 volumes of ethylenglicol, 4 volumes of PB 0.1 M) for long-term storage. A list of primary antibodies for stain NE tissue is described in the Additional file 5: Table S5. Images were obtained with a Zeiss LSM 510 confocal microscope and processed using Fiji. Cell quantification was done by *t*-test analysis using GraphPad Prism7. Data were expressed as mean ± standard error of the mean (SEM) of at least five independent slices analyzed in which fifteen cells were counted.

### Axonal length

*Z*-Axis stacks from entire monolayer cells were collected by confocal microscopy as was previously mentioned. After, images were imported to IMARIS 3D software v6.3.1 (Bitplane Sci Software, Zurich, Switzerland). Quantification of axonal length was performed using measurement point function of IMARIS follow the 3-dimensional trajectory of axons in *Z*-axis. Trajectory of neurites was tracked from the beginning to the end of process as described previously [15].

### Embryoid body formation

To assess the differentiation capability of the hiPSC cells a spontaneous differentiation protocol was performed adapted from [16]. Briefly, hiPSC were mechanically detached and maintained in non-adherent plates in EB differentiation media [DMEM high glucose (Invitrogen) supplemented with 20% of Bovine fetal serum (GIBCO), 2 mM Glutamine (SIGMA), 0.3 ug/ml Amphotericin B (Invitrogen), 100 U/ml Penicillin (Invitrogen), 100 ug/ml Streptomycin (Invitrogen)]. Media was changed by decantation every 48 h during the next 7 days. After that period EBs were attached in plates pretreated with 0.1% gelatin (SIGMA) until final differentiation (day 20). EB pellet Samples were taken at day 4, 7 and 20, as well as hiPSC colonies. Total RNA was extracted from pellet samples (Genelute Mammalian Total RNA Miniprep Kit, Sigma), and retro-transcribed (Superscript II, Invitrogen). Antibodies used to characterize the EB are listed in Additional file 6: Table S6 and Additional file 7: Table S7.

### FGD6 sequence confirmation in hiPSC and differentiated cells

Using as starting material total RNA extracted with RNeasy Mini Kit (QIAGEN) the complementary DNA strand was synthetized with SuperScript III and oligo(dT) primer. The region of interest was amplified with an AccuPrime Taq DNA polymerase High Fidelity (Invitrogen) and specific primers (Additional file 8: Table S8). The program was 1 cycle at 94 °C for 1′, 35 cycles at 94 °C for 30″, 63–57 °C or 55 °C for 30″ and finally 68 °C for 1′. The amplification product was sent to for sequencing and the result was analyzed using the program Clampex.

### Neuroepithelial differentiation

The protocol was adapted from [17–19]. Briefly, hiPSC colonies were mechanically detached and transferred to a non-adherent plate with hiPSC media without FGF for 5 days with media change every 48 h by decantation. After that period the media was changed for neural induction media 1 (NIM1 suspension = DMEM F12, glutamine, amphotericin, NEAA, N2 1×, heparin 2 ug/ml). At day 8 the aggregates were transferred to adherent plates pretreated with gelatin 0.1% and SFB. The attached aggregates were further maintained for 10 days in NIM1 1st adhesion to obtain primitive NE (NIM1 1st adhesion = NIM1 suspension + BMP2 2uM Sigma SB431542). At day 17 radial arrangements of columnar cells that surround a central lumen that arise stochastically in monolayer cultures of hiPSC upon neural induction, and are thought to mimic the morphology of a cross-sectional slice of the embryonic neural tube are manually selected and placed in a new non-adherent plate for 8 days in NIM 2nd suspension (NIM 1st suspension, 1uM cAMP, B27 1X, IGF-1 10 ng/ml). At day 25 those aggregates were placed onto polyornitine/laminin-pretreated plates in neural differentiation media (NDM = Neurobasal:DMEMF12, glutamine 2 mM, amphotericin, NEAA0.1 mM, N2 1×, B27 1×, cAMP 1uM, ascorbic acid 10 ng/ml, BDNF 10 ng/ml, GDNF 10 ng/ml, IGF-1 10 ng/ml).

### Electrophysiological recordings

For electrophysiological recordings NE attached to coverslips were transferred to a perfusion chamber perfused with ACSF (in mM: 125NaCl, 2.5KCl, 1.3NaH_2_PO_4_ · H_2_O, 26NaHCO_3_, 2CaCl_2_, 1MgCl_2_, 10glucose) at a constant flow (2 ml/min). Visualization was performed using an upright microscope (Nikon Eclipse) equipped with an immersion objective 40×, DIC optics and an infrared camera connected to a monitor. Recording pipettes were made from borosilicate capillary glass (Sutter 150 110 10) using a pipette puller (P-97, Sutter). The pipette was filled with a solution containing (in mM) 20 KCl, 120K-gluconate, 10 HEPES, 3Na_2_ATP, 0.3 NaGTP, 0.1 EGTA, 10 phosphocreatine and 2 MgCl_2_, adjusted to pH 7.3 with KOH. The signal was acquired with an Axopatch-1D amplifier (Molecular Devices), digitized at 20 kHz (Digidata 1322A, Molecular Devices), and acquired using a PC with 9.2 pClamp acquisition program.

**Fig. 1:**
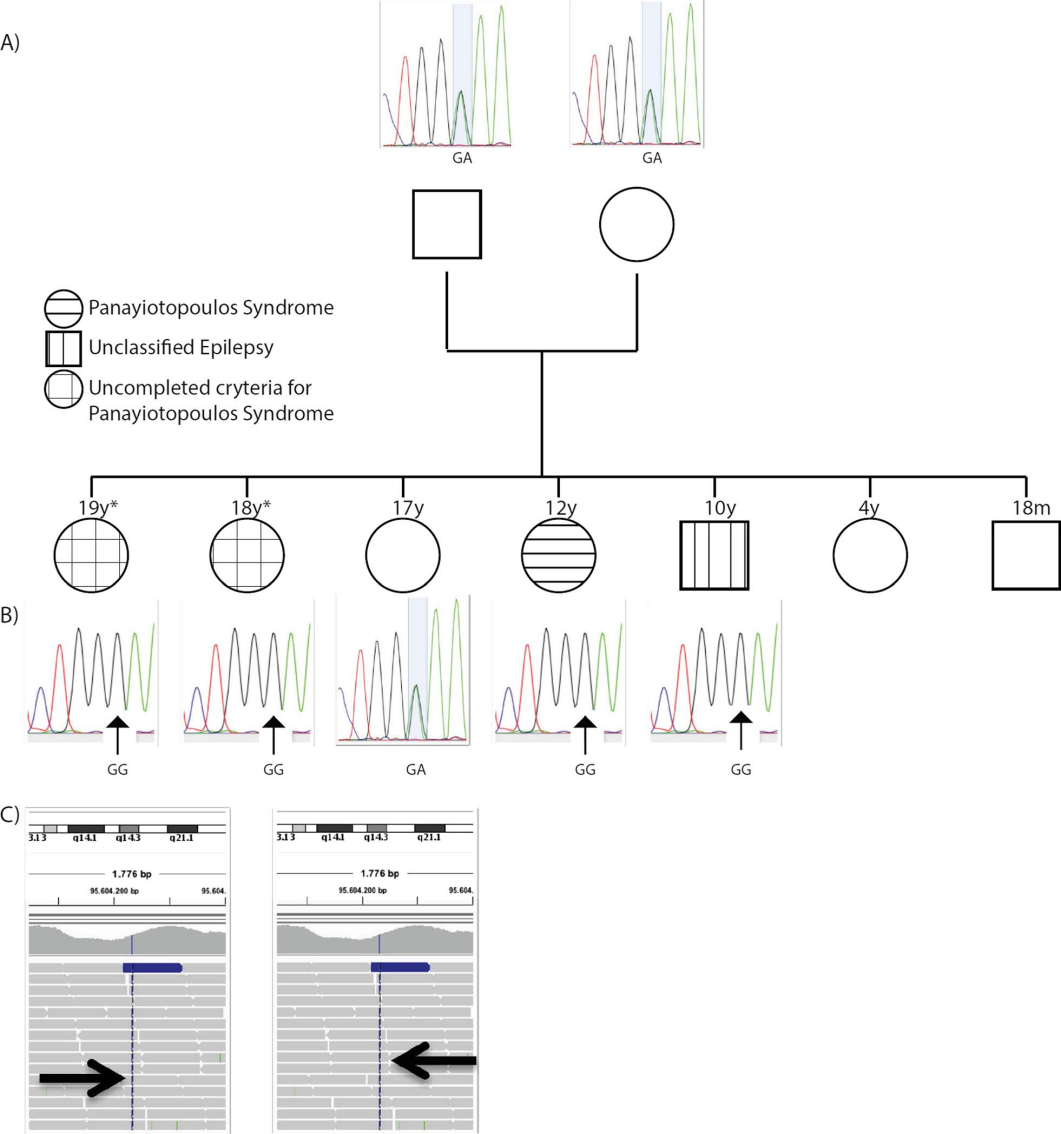
Pedigree of the family studied. **a** Four siblings suffering from focal epilepsy with onset in the first decade of life are indicated with different patterns indicating variability in epileptic phenotype. **b** Sanger sequencing of FGD6 p.E276G variant, where it is observed heterozygosity in asymptomatic siblings and parents and homozygosity in symptomatic siblings. **c** Representative capture of WES alignment where the FGD6 variant was first identified in both elder symptomatic siblings

The cells recorded for analysis were selected by morphology (large oval or pyramidal cell body with processes, Fig. 1a) and located in the periphery of organoids for ease of access. Only cells with a resting membrane potential of at least − 45 mV were used for analysis. Data analysis and measurements were performed with Clampfit (pClamp 9.2 software, Axon). Input resistance was determined as Δ*V*/Δ*I* at steady-state, where Δ*V* is the change in membrane potential produced by a hyperpolarizing current pulse of − 30 pA (Δ*I*) and 150 ms. The steady-state was estimated from an exponential fit to the voltage trace during the pulse. The threshold for action potentials was determined from action potential phase plots as the point at which the derivative of the voltage trace with respect to time (d*V*/d*t*) deviated from the mean baseline value by > 2 standard deviations [20]. Action potential width was measured at 80% of maximum amplitude. Capacitance and series resistance was compensated at least 80% in voltage clamp recordings. Settings were determined by compensating the transients of a small (10 mV) 50-ms hyperpolarizing voltage step. Leak conductance was not subtracted. Resting potential was measured in current clamp mode. For statistical analysis, GraphPad Prism version 5.00 for Windows (Graph-Pad Software) was used. The statistical differences were established using two-way ANOVA with Bonferroni post-hoc tests. The significant *p* value was set at 0.05 and all data are expressed as mean ± SEM unless otherwise specified.

### Gene expression analysis

RNA was extracted from 10 week-NE from patients and controls as described [21]. Functional genomics analysis was performed by hybridizing these RNAs to a 4 × 44 Human Agilent Array, containing 44,495 probes, representing 21,771 unique genes. Quality analysis of gene expression levels/chip (background levels, peak distribution, positive and negative probes distribution), normalization and quality assurance of the experiment were performed before differentially expressed genes were identified. The False Discovery rate and the LIMMA library were applied [22–24].

## Results

### Identification of a p.E276G homozygous variant in the FGD6 gene as a putative contributor to the underlying panayiotopoulos syndrome (PS), a form of occipital SLCE

We have sequenced the exome of two patients of a family in which 4 of the 7 members presented a diagnosis of occipital SLCE, with symptoms suggestive of PS (Fig. 1a, Additional file 1: Table S1). Using criteria described in Materials and Methods, we have detected about 75,000 single nucleotide variants and 8000 indels in each patient. More than 99% of these variants have been previously described in the literature or in databases with a frequency greater than 5% or localized within non-coding or regulatory regions, diminishing to a minimum their pathogenic potential. Finally, of the 47 genes with variants consistent with a recessive model, we have identified only the variant p.E276G in homozygous form in the FGD6 gene as a probable contributor of the phenotype observed in this family, showing perfect segregation with those family members affected by the disease (Fig. 1b, c).

### Generation of hiPSC-derived neuroepithelium from 2 patients and 2 unaffected family members as controls

All procedures were approved by the IRB of the Ramos Mejía Hospital and all participants in the study have signed an informed consent. Skin biopsies were harvested from 2 patients (P1 and P2) and 2 unaffected family members (mother and sister) as controls (CR1 and CR2) (Fig. 1). Fibroblasts were successfully obtained from all explants with more than 90% homogeneity as assessed by immunofluorescence with an anti-fibroblast specific antibody (Additional file 9: Fig. S1). hiPSC clones were generated from all derived fibroblasts using the STEMCCA vector [11] driving the expression of octamer-binding transcription factor 4 (*OCT4*), (sex-determining region Y)–box 2 (*SOX2*), Kruppel-like factor 4 (*KLF4*), and myelocytomatosis viral oncogene (*c-MYC*). Healthy hiPSC colonies (flat compact looking cell clump with defined edges composed by cells with big round nuclei and nucleolus with small cytoplasm) were selected by morphology and tested positive for markers of pluripotency including the proteins homeobox transcription factor Nanog, podocalyxin-like protein 1 (TRA1/80), sex determining region Y-box 2 (SOX2) and octamer-binding protein 4 (OCT4) by immunofluorescence and mRNA expression of *OCT4, SOX2*, Nanog, hylopetes phayrei microsatellite DNA locus hph34 (*HPH34*), zinc finger protein 42 homolog (*hREX*) and megalobrama hoffmanni GDF34 microsatellite (*hGDF34*) (Additional file 10: Fig. S2A). Transgenes insertion and expression in hiPSC lines were also confirmed by PCR and RT-PCR (Additional file 10: Fig. S2B). All hiPSCs present a normal karyotype, with one exception (Additional file 10: Fig. S2C). Clone F14A141 from a control subject presented a balanced translocation between chromosome 3 with a cut point at band p21.3 and the p arm of chromosome 15 (cut point at p11). Since this translocation is balanced, there is not net gain or loss of genetic material and it is expected that these cells should not gain or lose any functions and therefore, their phenotype should not be affected as shown before [25]. hIPS clones show silencing of the transgenes after continuous passages in several lines as expected (Additional file 10: Fig. S2D). The differentiation potential of hiPSC clones was tested by embryoid body (EBs) formation. To allow spontaneous differentiation of hiPSC, clones were cultured for a period of 20 days with 10% of fetal bovine serum first in suspension and then in adhesion culture. We consistently observed the presence of spontaneous beating bodies by 20 days in vitro (div) indicating successful differentiation (data not shown). mRNA expression of lineage specific markers (endoderm: alpha fetoprotein (*AFP*), chemokine receptor type 4 (*CXCR4*); mesoderm: Brachyury, platelet endothelial cell adhesion molecule (*PCAM1*); ectoderm: microtubule associated protein 2 (MAP2), and paired box protein pax-6 (*PAX6*) were tested at 0, 4, 7 and 20 days after EBs formation (Additional file 10: Fig. S2E, left) showing a pattern of expression consistent with a spontaneous differentiation process. As control, pluripotency marker OCT3/4 mRNA was also quantified during the differentiation process showing decreased levels until a complete loss by day 20. End point differentiation (20div) protein expression by immunocytofluorescence (ICF) also showed the presence of lineage specific markers for the three lineages (endoderm: AFP; mesoderm: Troponin, Desmin; ectoderm: PAX6, class III β tubulin (TUJ), doublecortin (DCX), SOX2) (Additional file 10: Fig. S2E, right). hiPSC clones were stable in culture for more than 20 passages. These data confirmed the successful generation of *bona fide* hiPSC cell lines.

**Fig. 2:**
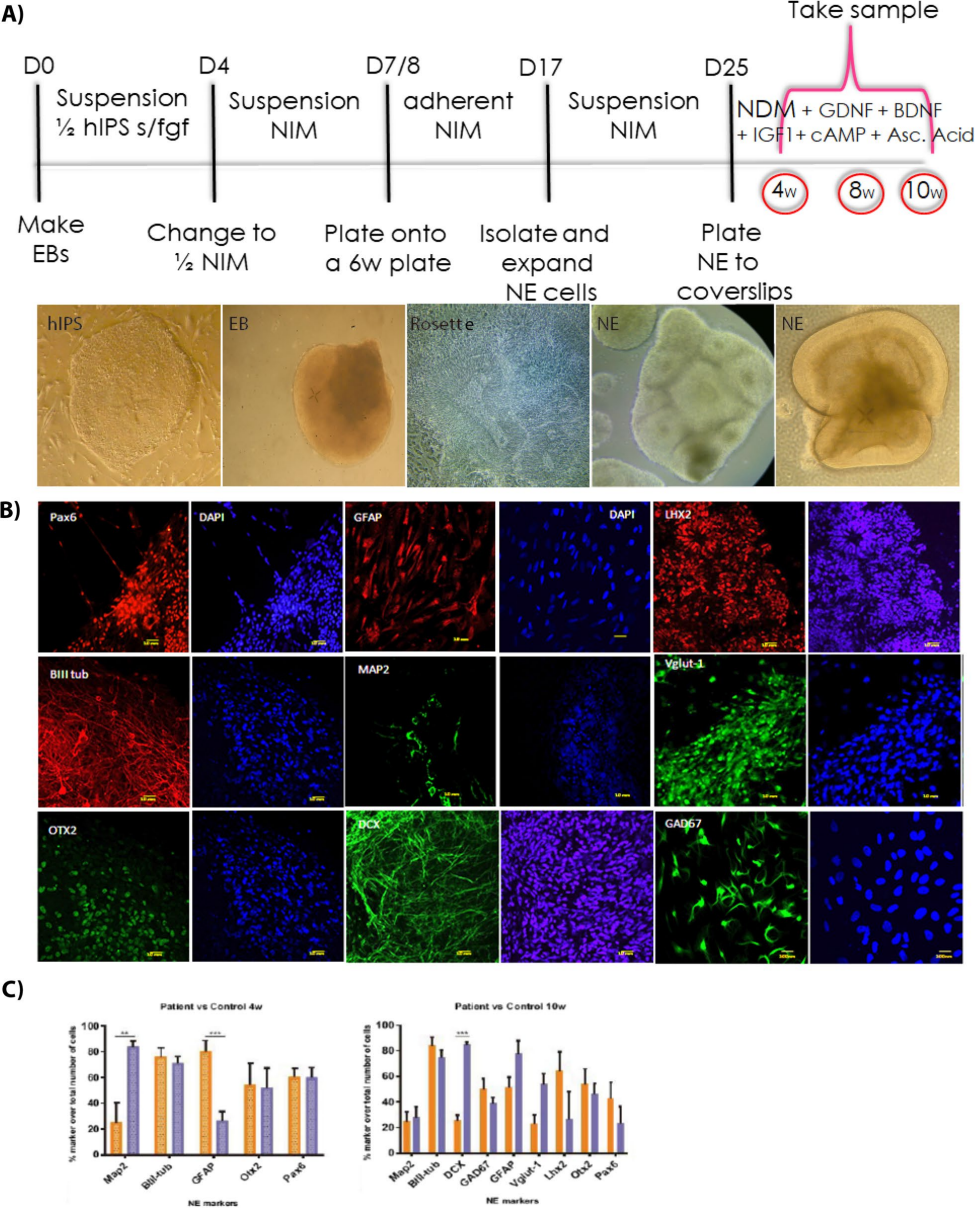
Neuroepithelial derivation and characterization. **a** Schematic timeline and images of NE derivation. During the first 8 days, hIPS are induced to form EBs in suspension with hIPS media without FGF during the first 4 days and then switch to NIM. At day 8 the EBs are plated to form neural rosettes (middle image). At day 17, rosettes are manually selected and further cultured to obtain mature NE until the desire point of maturation. **b** Immunocytofluorescence of NE cultures showing immature (GFAP, DCX, LHX2, OTX2, PAX6) and mature neuronal markers (BIII Tub, MAP2, Vglut-1, GAD67). **c** NE markers quantification at 4 (Map2_*p*<0.0038_, BIII tub, GFAP_*p*<0.0002_, Otx2 and Pax6;) and 10 (Map2, BIII tub, DCX_*p*<0.0003_, Gad67, GFAP, Vglut-1_*p*<0.019_, Lhx2, Otx2 and PAx6) weeks of differentiation. Data was analyzed by Two-way ANOVA with Bonferroni Test

Neuroepithelial (NE) tissue was then derived from clones verified for differentiation and pluripotency capability (Fig. 2a). As expected, the composition of the NE tissue was heterogeneous, comprising progenitors implicated in neocortex development [PAX6^+^, Lim homeobox 2^+^ (LHX2^+^) and orthodenticle homeobox 2^+^ (OTX2^+^)]; migrating young neurons (DCX^+^); mature neurons (βIIItub^+^ and MAP2^+^) and type-specific inhibitory (glutamate decarboxylase 1^+^ (GAD67^+^) and excitatory [vesicular glutamate transporter^+^ (VGLUT-1)] neurons as well as putative astrocytes or neural progenitors (GFAP^+^) (Fig. 2b). We next performed a thorough, quantitative analysis of the expression of neural markers in these cells at 4 and 10 weeks of NE differentiation. Figure 2c shows that most neural markers were expressed at similar levels in both the mutant and normal cell populations at both time points studied. However, at 4 weeks, GFAP expression was significantly higher (*p* < 0.0002) and MAP2 levels were significantly lower (*p* < 0.0038) in the patient-derived NE cells compared to the controls. In addition, the expression of DCX was significantly lower (*p* < 0.019) in patient-derived NE than in controls at 10 weeks. All other 8 markers studied, including β-III tubulin which is a robust marker of neuronal cells, did not show any significant difference between both populations at any time point. While we could not discard minor differences in cell composition, we conclude that, overall, there were no major shifts in cell composition of patient and control-derived NE using this technique.

**Fig. 3:**
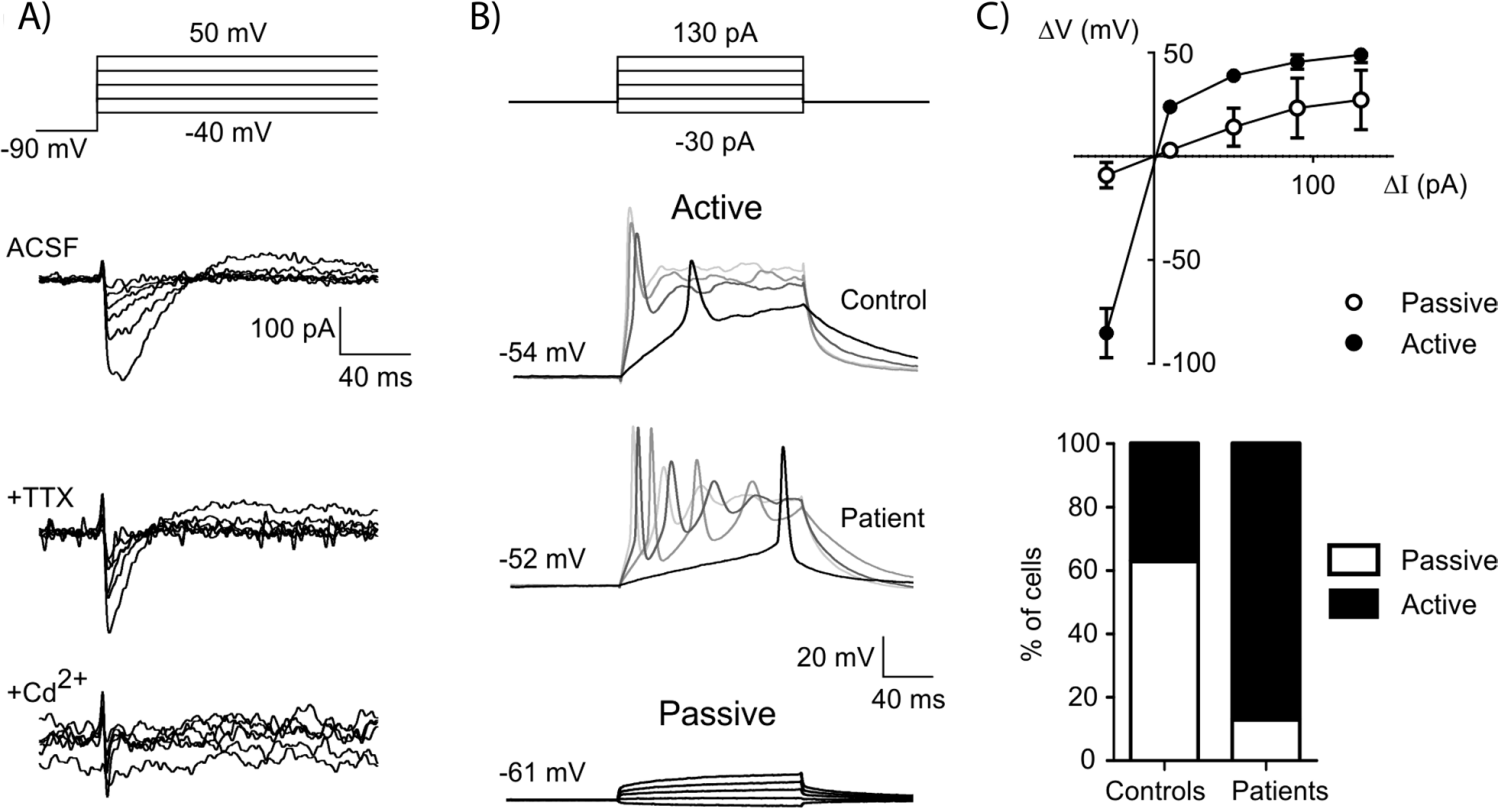
Representative voltage clamp recordings of a cell from NE **a** NE cells display fast inward currents with sensitivity to TTX (10 µM) and cadmium (100 µM) when subjected to the series of voltage steps schematized above. **b** representative current clamp recordings of two active cells recorded from control and patient NE as indicated, and a passive cell recorded from control NE, in response to the series of current steps schematized above (12 weeks in culture). **c** The top graph shows average voltage vs. current curves for passive and active cells from control NE. The bottom graph shows the percentage of active and passive cells found in NE of control and patients at 12 weeks in culture. Significantly different, Chi Square (*p* < 0.04, number of observations: 8 cells/group)

Then, we carried out whole cell patch clamp recordings to evaluate the functionality of the NE cell population. Cells recorded from NE tissue presented functional voltage-gated Na^+^ and Ca^2+^ channels as evidenced by the presence of inward Na^+^ (TTX-sensitive) and Ca^2+^ (CdCl_2_-sensitive) voltage-gated currents (Fig. 3a). Active cells displaying action potentials were observed in NE from both patients and control subjects (Fig. 3b–c).

### Neuroephitelial cells from patients develop shorter axons and show dramatically reduced levels of synapsin-1

**Fig. 4:**
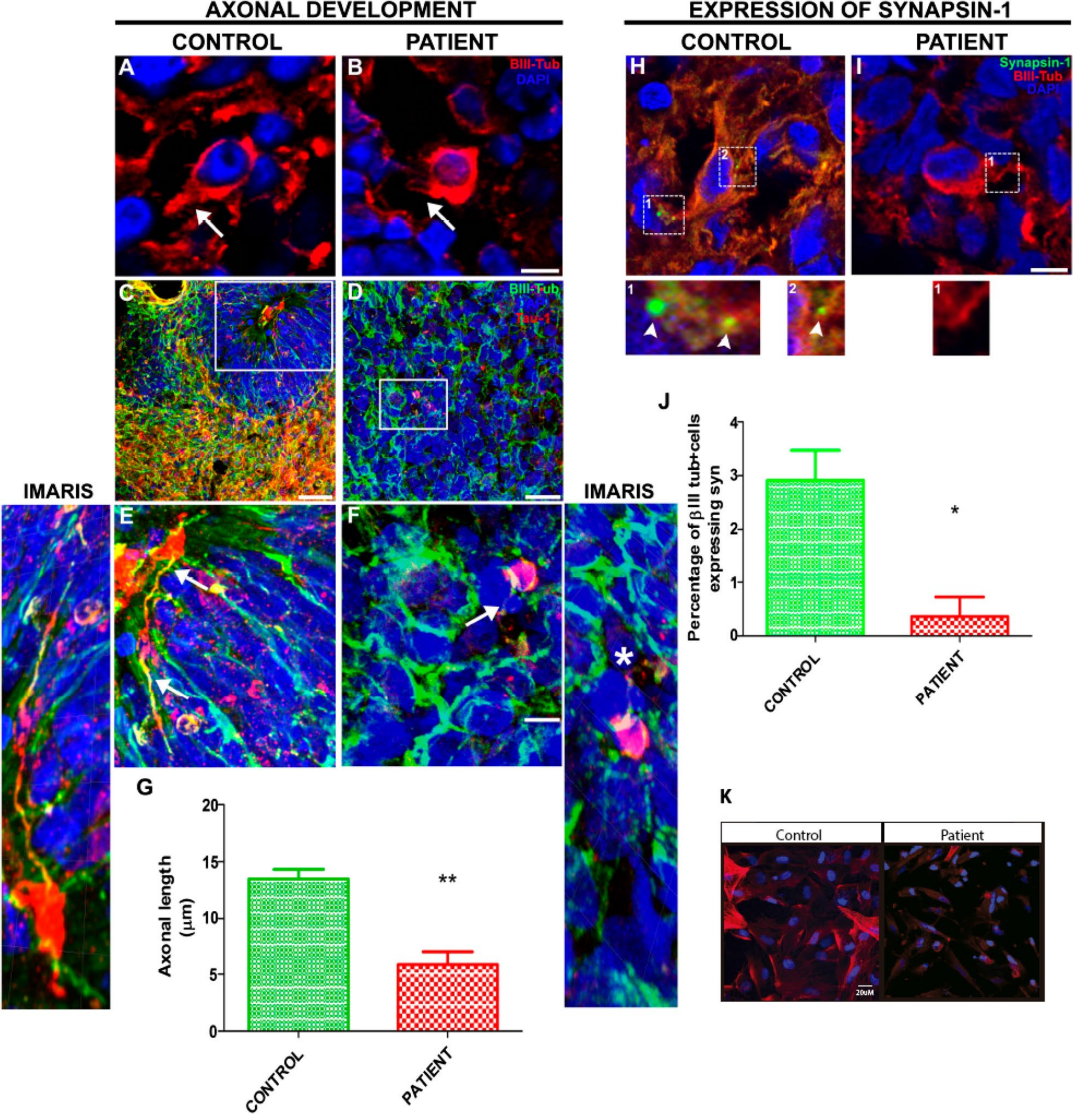
Analysis of axonal length and synapsin-1 expression. Left panel: axonal length in neurons derived from controls and patients. **a**, **b** Tuj-1b (red) + Dapi (blue); **c**–**f** Tau-1 (red) Tuj-1b (Green) Dapi (blue). Side insets show the 3D-trajectory of axons in IMARIS images. White arrows show axonal initial segments as well as axon distal segment; the asterix show truncated neurite. **g** Quantification of axonal length (µm) for Tau-1 positive neurons derived from controls and patients at 7 weeks of differentiation **p = 0.0064, *t* test, 2 tail. Right panel: expression of Synapsin—1 clusters in neurons derived from controls (**h**) and patients (**i**). The inset corresponding to the magnification of clusters are shown in lower panels. **j** Quantification of the synapsin − 1 clusters in BIII-tubulin positive cells (Tuj-1b) *p = 0.032, *t* test, 1 tail. **k** Arrangement of microfilaments by phalloidin staining. Bar scale. 20 microns

By structural analysis, FGD6 protein presents features related with Rho protein activation which is known to play a crucial role in neuronal morphology and synaptogenesis [26–28]. For that reason, we next analyzed axonal length and presence of synaptic vesicles in the derived NE. Double labeling with the neuronal marker β-III tubulin and the axonal marker Tau-1 or the synaptic vesicle marker synapsin-1 (SYN-1) were used. Neurons presented long, thin and defined processes. The average nucleus size was 8.5 microns with round and small cell body (data nor shown). Qualitative evaluation of cultures suggested a much lower amount of neurons with defined processes in the NE derived from epileptic patients than in control cells (Fig. 4a–f). When axonal length was measured, a dramatically reduction in the patient’s derived neurons was observed when compared to controls (*p* < 0.0064, three independent slices were analyzed in which at least six cells were counted) (Fig. 4g).

Similarly, the amount of SYN-1 positive clusters found in neurons derived from epileptic patients was reduced when compared to controls (*p* < 0.032) (Fig. 4h–j). We concluded that patient-derived cells showed shorter axons and diminished number of synaptic vesicles compared to controls.

In addition, we studied the state of the cytoskeleton, in particular the arrangement of microfilaments using phalloidin staining. Patient-derived cells presented a disorganized cytoskeleton, showing signs of aggregation compared to controls (Fig. 4k).

### Functional analysis at the electrophysiological level

**Fig. 5:**
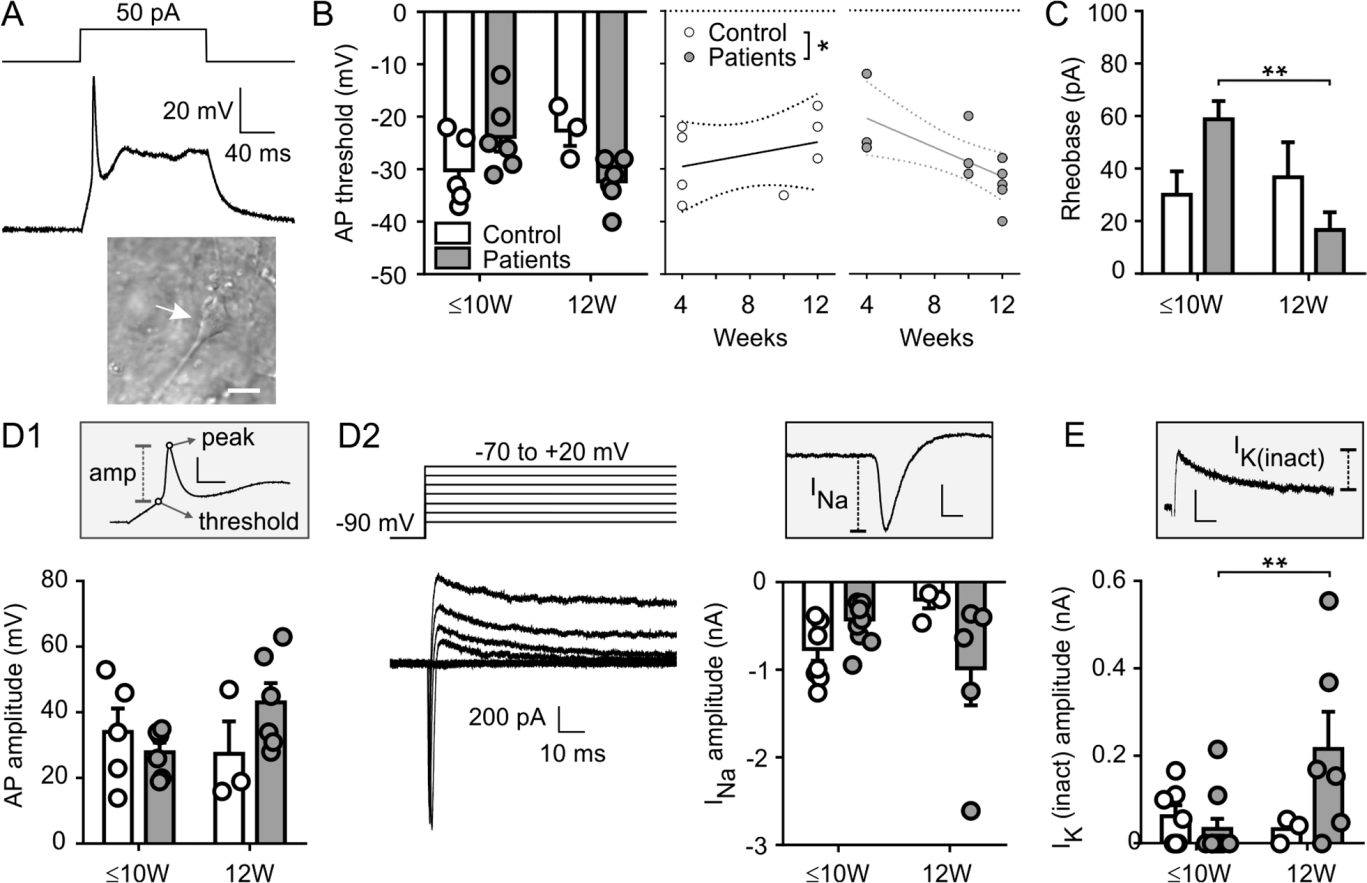
Electrophysiological properties of active cells recorded from cultured neuroepithelium. **a** Representative voltage response of a control cell to a 50-pA depolarizing step (top) and representative DIC image of a recorded cell (bottom); scale bar: 50 µm. **b** Left, average threshold for the first action potential elicited by a 50-pA pulse; significant interaction after 2-way ANOVA with disease and time as factors (*p* = 0.0110); right, linear regression analysis (threshold vs. time of differentiation) showing a different developmental trajectory for control and patient NE (significantly different slopes, *p = 0.0244; slope significantly different from zero for patients, *p* = 0.0181). **c** Minimum depolarizing current amplitude that elicited an action potential (rheobase) after a series of depolarizing steps (− 30 to 130 pA, Δ40 pA) **p < 0.01 (Bonferroni multiple comparisons after significant interaction in two-way ANOVA). **d1** Amplitude of the first action potential elicited by a 50-pA pulse, measured as indicated in the inset (scale: 20 mV, 10 ms). **d2**, Representative traces of whole-cell currents elicited by a series of voltage steps (− 70 to + 20 mV, Δ15 mV) from a pre-pulse potential of − 90 mV. The graph shows values for amplitude of the fast inward current measured in response to a voltage step to − 5 mV, as indicated by the inset (scale: 500 pA, 1 ms). **e** Amplitude of the fast inactivating outward current measured in response to a voltage step to − 25 mV, as indicated by the inset (scale: 200 pA, 10 ms). Data shown as Mean ± SEM with data from individual cells as symbols (Control in white and patients in gray) and analyzed with two-way ANOVA (factors were disease: control vs. patient and time: ≤ 10 weeks vs. 12 weeks). Number of observations were: In **b**, **c** and **d1** ≤ 10 W, 6 cells/2 patients and 5 cells/1 control; 12 W, 6 cells/2 patients and 3 cells/1 control; In **d2** and **e** ≤ 10 W, 8 cells/2 patients and 7 cells/1 control; 12 W, 5 cells/1 patient and 3 cells/1 control

To determine whether the NE neurons derived from patients and controls were functionally different, we did whole cell current and voltage clamp recordings at different times of the differentiation process. Cells were selected by shape (large oval cell body with processes) and location (in the periphery of organoids and adhered to coverslip). The probability of finding excitable cells was higher in epileptic patients at 12 weeks in culture than in controls (*p* < 0.001, Chi-square) (Fig. 3b, c). We found no differences between groups in resting membrane potential of active cells (− 55 ± 3 mV vs. − 51 ± 4 mV at ≤ 10 weeks and − 48 ± 2 mV vs. − 55 ± 3 mV at 12 weeks, when comparing patients with controls, respectively; two-way ANOVA, *p* > 0.05, n = 3–8 cells per group). However, during depolarizing current injection the NE cells derived from patients developed a lower action potential threshold with time of in vitro differentiation, which was not observed for control cells (Fig. 5a, b). Additionally, the amount of current needed to elicit an action potential (rheobase) was smaller in cells recorded from NE derived from patients at 12 weeks of differentiation when compared with shorter times in culture (Fig. 5c *p* < 0.01). These changes cannot be attributed to changes in the Na^+^/K^+^-mediated action potential, as evidenced by similar action potential amplitudes and fast inward currents in patient and control cells (Fig. 5d). Action potential duration and latency were also similar in both groups (Additional file 11: Fig. S3A). Interestingly, more than half the cells recorded from patients at 12 weeks of differentiation displayed a repetitive action potential discharge pattern that was not observed in control cells at this time point (Additional file 11: Fig. S3B). Overall these results indicate an increased excitability in patient cells that emerges with the time in culture.

The mechanisms underlying this enhanced excitability remain unclear. Although the recorded cells had a high input resistance in both groups, patient-derived cells had a significantly lower input resistance than control-derived cells (*p* < 0.05) (Additional file 11: Fig. S3C). Whole cell voltage-clamp experiments showed similar amplitude of delayed non-inactivating outward potassium currents regardless of degree of differentiation (≤ 10 and 12 weeks) or medical condition (patient vs. control) (Additional file 11: Fig. S3D). However, a fast inactivating outward potassium current was of higher amplitude at 12 weeks of differentiation in samples from patients (*p* < 0.05) (Fig. 5d). The enhancement of this current could facilitate repetitive spiking in cells from patients (Additional file 11: Fig. S3B).

### Functional genomics analysis shows a tendency towards immaturity in patient-derived cells

**Fig. 6:**
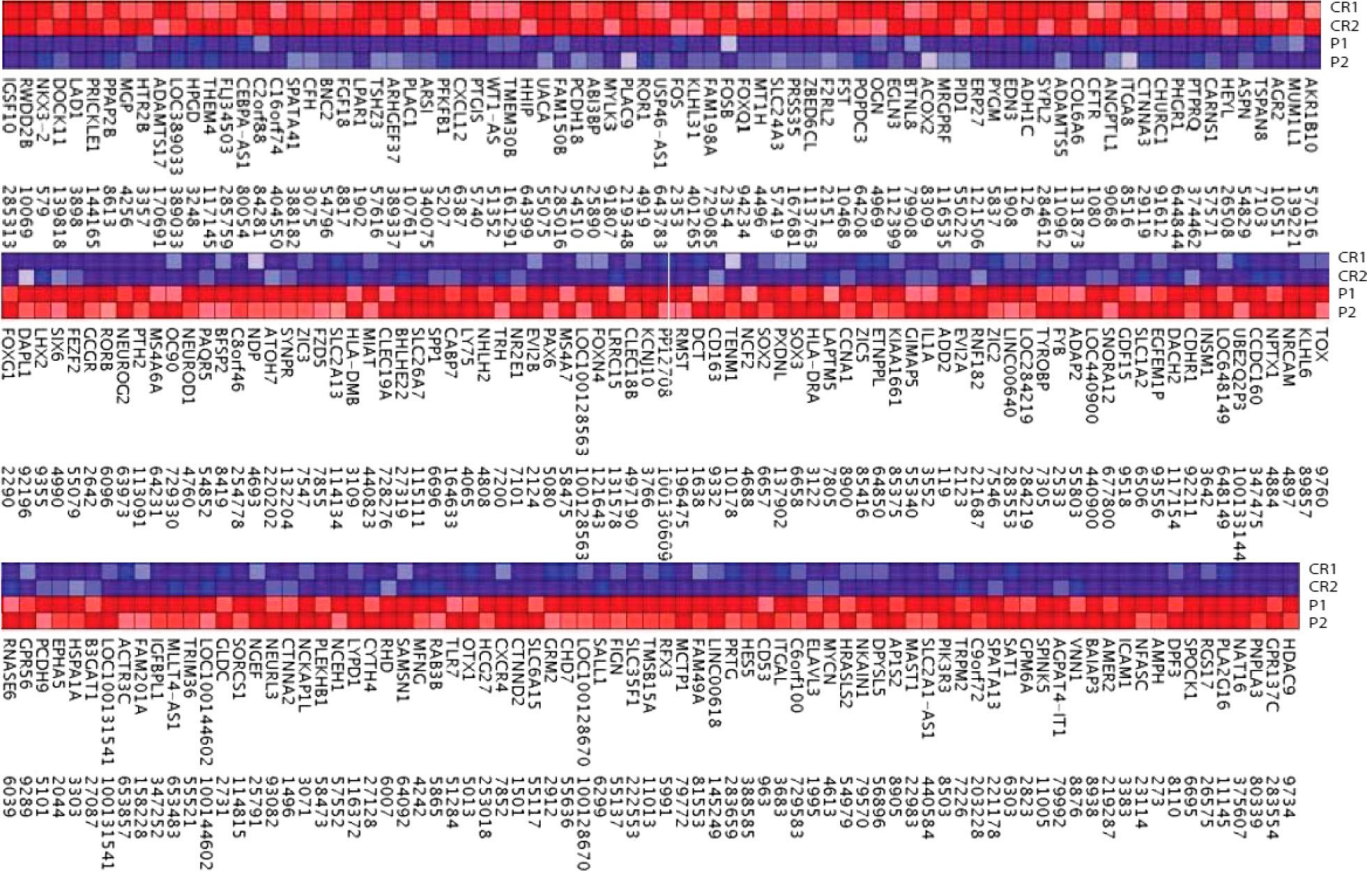
Functional genomic analysis in patient (P1, P2) and control (CR1, CR2)-derived neuroepithelial tissue. 194 differentially regulated genes were detected (upregulated genes are shown in red (123) and downregulated genes in blue (71)

Next, we performed functional genomics analysis of patient and control NE cells (Fig. 6). This analysis led to the identification of 194 differentially expressed genes, 71 of which were downregulated and 123 upregulated in patient-derived NE cells, compared to controls. Gene Ontology analysis showed a tendency towards neuronal immaturity in patient-derived cells since several differentiation-related transcripts were over-represented in these cells. For example: Regulation of cell differentiation (*FGF18, NEUROG2, FOXG1*), regulation of neurogenesis (*NEUROD1, PAX6, SOX2, SOX3, FOXG1*), positive regulation of neural cell precursor proliferation (*DCT, PAX6, SOX2*), positive regulation of cell differentiation (*NEUROD1, CXCR4, FOXG1, FEZF2*) and positive regulation of neurogenesis (*NEUROD1, NEUROG2, PAX6, FEZF2*). Therefore, the functional genomic pattern obtained is consistent with a delay in maturation in hiPSC-derived neurons from patients.

## Discussion

We have studied a family of 7 siblings where 4 of them were affected by occipital SLCE. We have obtained and thoroughly characterized NE derived from *bona fide* hiPSC cells from SLCE patients and familial controls. The electrophysiological recordings show that control NE samples contain maturing neurons with relatively slow action potentials compatible with previous reports [29, 30]. We have observed that NE cells express neuronal markers and were electrophysiologically active. Patient-derived NE neurons possessed shorter axons, a disorganized cytoskeleton, fewer synaptic connections and an enhanced excitability compared to control-derived NE cells. These differences arose with time in culture, together with a gene expression profile biased towards an immature phenotype. We conclude that the in vitro model of SLCE provided evidence of distinct features related to neuronal immaturity in patient-derived-cells. In addition, this model could be used to more in-depth studies and can serve as a novel platform for drug screening.

Exome sequencing ruled out a role for well-known epilepsy-causing genes in this family. Noteworthy, among a list of 47 candidates, only a variant of unknown significance in FGD6 showed perfect segregation with all the affected members of this family leading us to hypothesize that it may have a role under a probable oligogenic model in the etiology of the epilepsy affecting this family. Nevertheless, the genetic architecture for occipital SLCE is still not completely understood [2]. Oligogenic inheritance has been proposed for SLCEs, complicating the recognition of etiological genetic abnormalities in single-family studies [31]. It is important to emphasize that our studies were not designed to functionally prove a pathogenic role of the discovered FGD6 variant [32]. Therefore, we herein describe this novel genetic finding and hypothesized its contributory etiologic role in the disease, for which studies in the future are warranted. Notwithstanding, FGD6 participation in the regulation of cell adhesion, cell polarity and membrane recycling through its interaction with actin-based protein networks has been previously described [33], building in the hypothesis that FGD6 regulates integrin interactions with F-actin mesh controlling actin polymerization and cytoskeleton dynamics. Moreover, FGD6 mutations have also been related to a subtype of macular degeneration and FGD6 downregulation leads to actin disorganization [34]. In line with previous reports on the functional effects of FGD6 loss of function, here we show a disorganized actin cytoskeleton in patient-derived cells. This observation correlates, at the neuronal level, with a reduced axonal length along with a lower number or synaptic vesicles. Similar cellular phenotypes have been described for mutation on the gene *PRICKLE*, which is associated with epilepsy [35]. *PRICKLE* organizes microtubule polarity and affects axonal growth influencing vesicle transport dynamics due to its association with synapsin-1 [36]. Synaptic GABAergic and glutamatergic reorganization not only change normal information processing but also facilitate seizure occurrence due to network alterations [37]. These data allow us to hypothesize that mutations in FGD6 could lead to impaired interactions of this protein with actin networks leading to disorganization of the actin cytoskeleton producing a shortage in axonal length that impacts vesicular trafficking with the subsequent impairment of synaptic connectivity that could contribute in addition to other unidentified genetic and non-genetic factors to the epileptic phenotype observed in SLCE patients.

DCX is the only marker to be differentially detected by immunofluorescence between control and patient NE after 10 weeks of differentiation. DCX is essential for stabilizing microtubules, nucleus-centrosome coupling during nuclekinesis and dynamically regulating the actin filament formation in developing neurons [38]. DCX binds directly to microtubules and interacts with F-actin through binding proteins to actin as spinophyllin. Thus, it is plausible to think that the observed deregulated expression of DCX might signal a role for abnormal neuronal migration in the pathophysiology of the disorder under study here.

The electrophysiological characterization showed that patient NE samples produced a higher proportion of active cells, which were more excitable. This enhanced excitability was observed as an enhanced response to current injection through the recording somatic electrode: less current was needed to induce action potential firing in patient-derived NE cells, which showed a lower action potential threshold. This phenotype emerged with time in culture and was associated to the divergent differentiation trajectories followed by control and patient NE. The emergence of hyperexcitability is frequently observed in developmental disorders of neural circuits and can obey to both cell-autonomous and non-cell-autonomous mechanisms [39–42]. Cell-autonomous defects are associated with dysfunction of membrane conductances that determine action potential discharge patterns [43, 44]. Non-cell-autonomous defects include aberrant mechanisms of communication among different cell types during circuit maturation [45, 46]. Our data support the hypothesis that both cell-autonomous and non-cell-autonomous mechanisms are involved in the aberrant hyperexcitability observed in patient NE cells. This hypothesis is based on the observations that the response of active cells to current injection through a somatic electrode (a measure of intrinsic or membrane excitability) was increased in patient NE cells, and that the emergence of synaptic markers was impaired in patient NE. A defect of potassium channel function or impaired potassium buffering from the extracellular milieu has been causally related to epileptic activity [44, 47], however, an exacerbation of potassium channel function can contribute to neuronal hyperactivity as well [48, 49]. Of particular interest is the emergence of repetitive discharge patterns and oscillatory activity when depolarizing and hyperpolarizing conductances of similar kinetics act in concert [50–52]. The enhanced fast potassium currents could contribute to a higher incidence of repetitive firing in patient NE cells [53, 54].

Gene expression analysis showed a tendency towards an enrichment of genes related to neuronal immaturity which was not previously observed by immunohistochemistry probably due to the sensitivity of the latter technique. These data are in agreement with the divergent maturation trajectories supported by the electrophysiological data and by the morphological results, and support the idea that patient-derived NE shows a phenotype of delayed maturation. Interestingly, this feature may explain the clinical presentation of the disease: in approximately half of the patients with SLCE, the symptoms disappear by the end of adolescent life [55]. The data presented here supports the hypothesis of an impaired neuronal maturation underlying the progression of the disease. Delayed maturation and increased excitability may appear as conflicting results. However, aberrant hyperexcitability has been linked to deficient and delayed synapse formation and with synapsin deficiency [15, 56, 57]. Whether the neuronal hiperexcitability shown here is secondary to delayed synapse formation remains to be determined.

Gene discovery outbreak led to more than 500 loci to be listed as potential genetic cause of epilepsy when mutated [58]. This along with the development of cell reprograming technologies [13] placed the in vitro modeling of epilepsies to be a revolutionary platform for mechanistic studies and drug discovery for precision therapy. Dravet Syndrome (SCN1A mutations) and Rett Syndrome (Mecp2 and CDKL5 mutations) are among the most studied syndromes related to epilepsy using this technology [59–66]. Altered synapse formation, reduced neurite outgrowth and spontaneous activity are the major characteristics described in these hiPSC-based epilepsy models. Similar to those models, we found reduced axonal growth and lower synaptic vesicles, more active and excitable neurons and a gene expression profile biased towards immaturity, a novel feature of neurons derived from epileptic patients.

In summary, here we described the first in vitro model of SLCE showing specific features that indicate a bias towards immaturity of patient-derived neurons and set the bases for further in-depth functional studies.

## Conclusions

Our results show patient-specific neuronal features reflecting immaturity, in resonance with immature images registered in the brains of this pediatric patients [6]. We are reporting the first in vitro model of SLCE which will enable further in-depth functional studies. Moreover, our approach using three dimensional cell cultures to further support neuronal differentiation and diversity can be valuable to link particular protein defects associated with cortical development, abnormalities which are usually seen in neurodevelopmental disorders like SLCE.

## Supplementary Information

**Additional file 1: Table S1.** Clinical features of siblings presenting with epilepsy.

**Additional file 1: Table S2.** Primers used to amplify bridge regions between genes found in STEMCCA.

**Additional file 1: Table S3.** Primers used to amplify endogenous pluripotency genes.

**Additional file 1: Table S4.** Primers list for EB lineage detection.

**Additional file 1: Table S5.** Primary antibody list to label pluripotency markers in IPS lines.

**Additional file 1: Table S6.** Antibody list to characterize neuroepithelial tissue.

**Additional file 1: Table S7.** Antibody list for EB lineage detection.

**Additional file 1: Table S8.** Primer sequence to detect FGD6 mutation in IPS and derived cell lines.

**Additional file 1: Figure S1.** Fibroblast characterization. Immunocytofluorescence of primary cell culture of fibroblasts obtained from skin biopsy (scale bar 10 μm).

**Additional file 1: Figure S2.** hiPSC characterization: pluripotency and differentiation. **A** hiPSC Pluripotency markers assessed by immunocytofluorescence (Nanog, TRA1-80, SOX2 and OCT4) (upper panel) and by RT-PCR (OCT4, SOX2, Nanog, HPS34 and hREX) (panel below) **B** Schematic representation of the STEMCCA (upper panel), PCR in hiPSC (lanes 1 to 8), fibroblasts (lanes 10 to 12) and STEMCCA 4x (lane 13) for OCT4-KLF4 and SOX-2-c-MYC bridges detection. **C** Full Karyotype of clones F13A282 P16 (P1), F9J143 P17 (P2), F12J121 P13 (CR1) and F14A141 P17 (CR2), by G-banding technique. P1, P2 and CR1 present a normal female karyotype, and CR2 shows a balanced reciprocal traslocation between chromosome 15 and 3 indicated by the dots (46,XX,t(3;15)(p21.3;p11)). **D** Representative RT-PCR of 5 clones (lanes 1-4, 5-8, 9-12, 13-16 and 17-20) to determine silencing of exogenous genes by detecting the expression of OCT4-KLF4 (lanes 1, 5, 9, 13 and 17) and SOX-2-c-MYC bridges (lanes 2, 6, 10, 14 and 18) in hiPSC lines. Lanes 3, 7, 11, 15 and 19: cyclophilin, Lanes 4, 8, 12, 16 and 20 are amplified samples with cyclophilin primers but no cDNA. Lanes 9 and 13 show non-silenced clones, as examples **E** EBs lineage specific detection by RealTime-RT-PCR (left panel) during the differentiation process (day 0, 4, 7 and 20) and immunocytofluorescence at day 20 of differentiation to detect the three lineages: endoderm (AFP) mesoderm (Brachyury, PECAM1, Troponin, Desmin) and ectoderm (PAX6,TUJ, DCX, SOX2) (scale bar 500um and 1mm).

**Additional file 1: Figure S3.** Electrophysiological properties of active cells recorded from cultured neuroepithelium. **A** Left, representative spike elicited by a depolarizing step of 50 pA in an active cell from neuroepithelium, indicating how latency and width were measured. Middle and right, action potential latency and width, respectively, measured for the first spike elicited in control and patient neuroepithelial active cells by a 50-pA depolarizing step when recorded at ≤10 and 12 weeks in culture. Not statistically different after two-way ANOVA. **B** Representative responses of active cells from control (left) and patients (right) to a 90-pA depolarizing step when recorded at ≤10 and 12 weeks in culture. The bars at the bottom represent the spike times of different cells after the pulse start (dashed line). Each row corresponds to a different cell. C, Input resistance of active cells recorded from patient- and control-derived cultures, significant main effect of disease (*p < 0.05). D, Left, representative current trace of an active cell in response to a depolarizing pulse to 25 mV from a holding potential of ‑90 mV indicating how potassium current amplitude was measured. Right, amplitude of delayed non-inactivating outward potassium currents for patient and control cells recorded at < 10 and 12 weeks in culture. Not statistically different after two-way ANOVA..

## Data Availability

All datasets used and/or analysed during the current study are available from the corresponding author on reasonable request.

## Abbreviations

SLCE: Self-limited childhood epilepsy
FGD6: FYVE, RhoGEF and PH domain containing 6 gene
hIPS: Human induced pluripotent stem cells
OCT4: Octamer-binding transcription factor 4
SOX2: Sex-determining region Y–box 2 (SOX2)
KLF4: Kruppel-like factor 4
c-MYC: Myelocytomatosis viral oncogene
Tra1/80: Podocalyxin-like protein
Hph34: Hylopetes phayrei microsatellite DNA locus hph34
hRex: Zinc finger protein 42 homolog
hGDF34: Megalobrama hoffmanni GDF34 microsatellite
EB: Embryoid body
AFP: Alpha fetoprotein
CXCR4: Chemokine receptor type 4
PCAM1: Platelet endothelial cell adhesion molecule
MAP2: Microtubule associated protein 2
PAX6: Paired box protein pax-6
TUJ: Class III β tubulin
DCX: Doublecortin
Lhx2: Lim homeobox 2
Otx2: Orthodenticle homeobox 2
GAD67: Glutamate decarboxylase 1
Vglut-1: Vesicular glutamate transporter 1
NIM: Neural induction media
NDM: Neural differentiation media
MAFs: Minor allele frequencies
