# Supplementary figures and images for: A familiar study on self-limited childhood epilepsy patients using hIPSC-derived neurons shows a bias towards immaturity at the morphological, electrophysiological and gene expression levels
Source: Stem Cell Res Ther. 2021 Nov 25;12:590. doi: 10.1186/s13287-021-02658-2 (PMC8620942; doi:10.1186/s13287-021-02658-2)

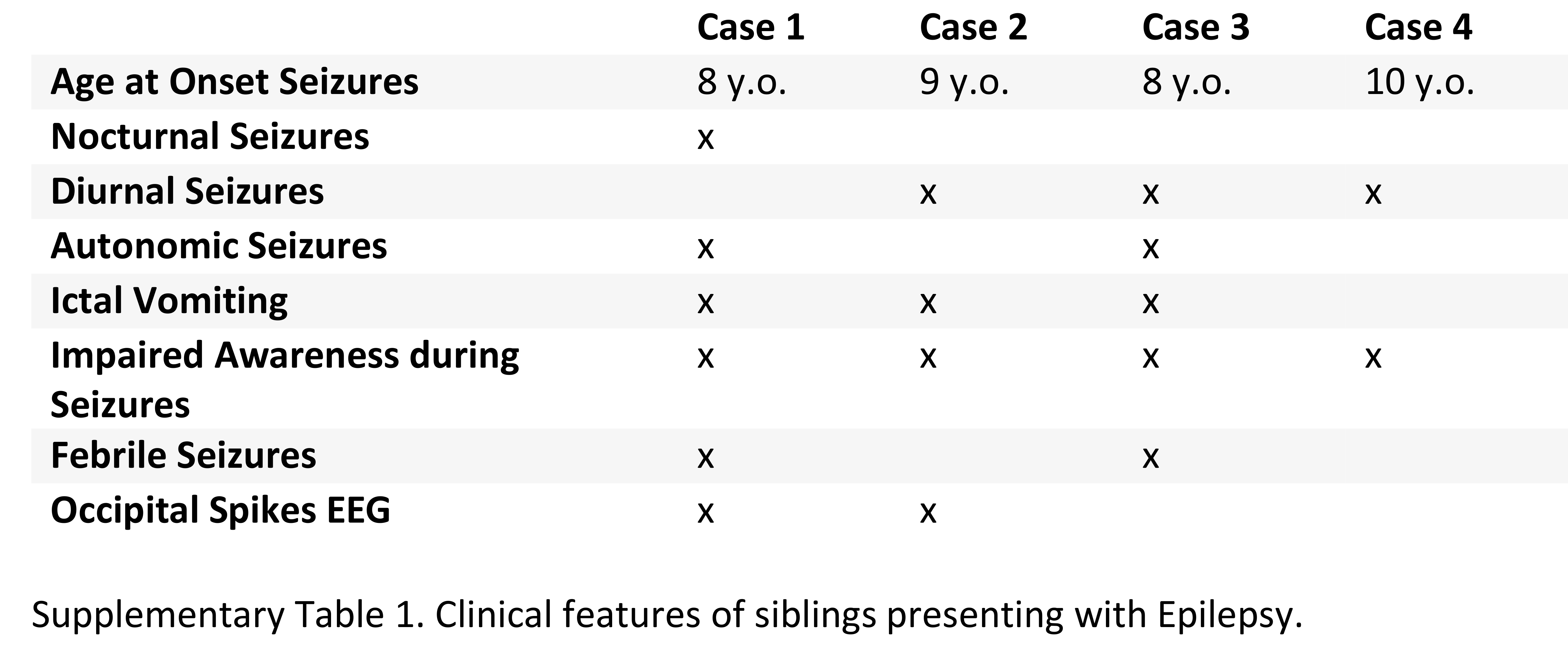

Supplement: Supplementary file 1 — Additional file 1: Table S1. Clinical features of siblings presenting with epilepsy. [file 13287_2021_2658_MOESM1_ESM.tif]

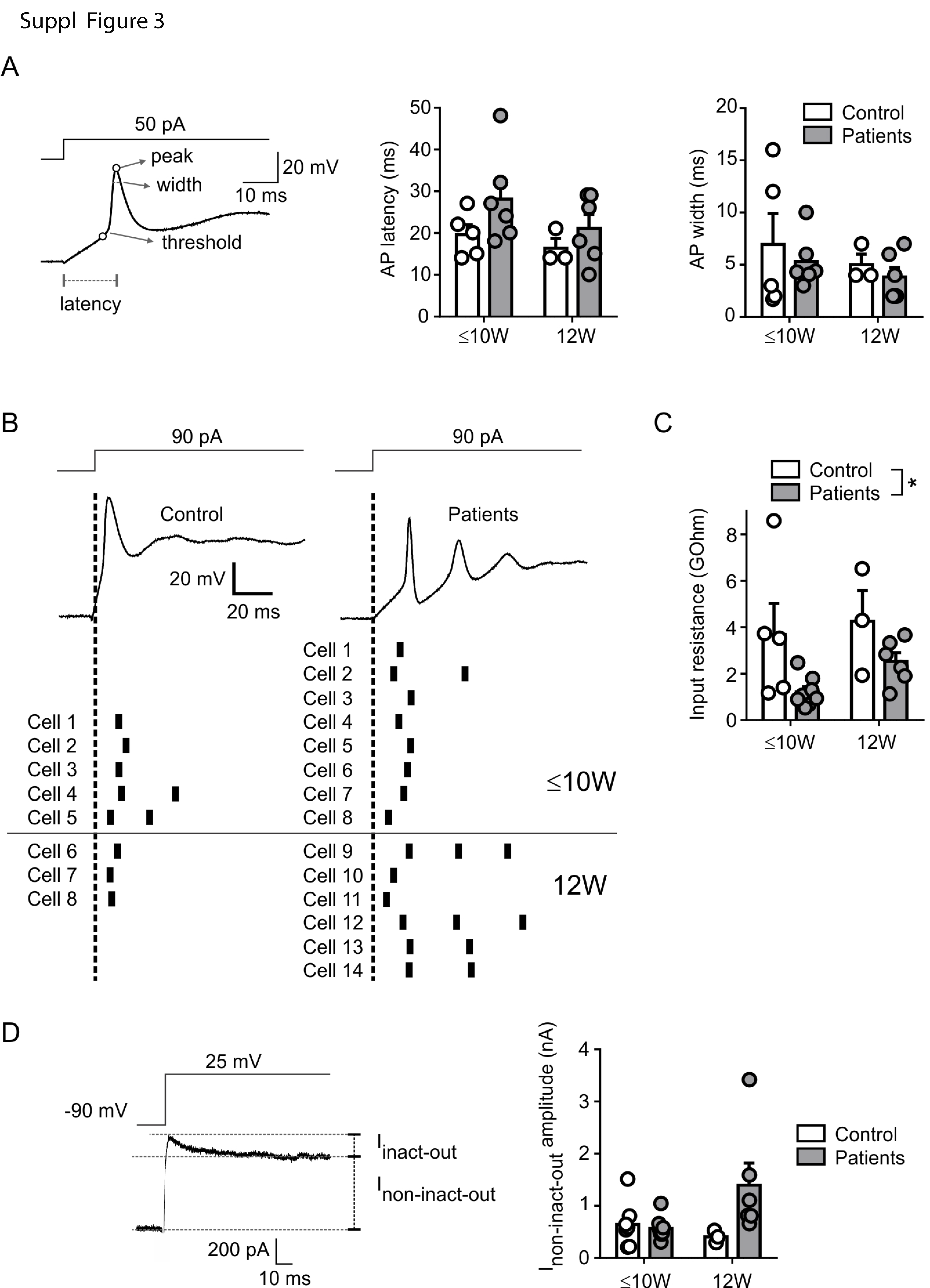

Supplement: Supplementary file 11 — Additional file 1: Figure S3. Electrophysiological properties of active cells recorded from cultured neuroepithelium. A Left, representative spike elicited by a depolarizing step of 50 pA in an active cell from neuroepithelium, indicating how latency and width were measured. Middle and right, action potential latency and width, respectively, measured for the first spike elicited in control and patient neuroepithelial active cells by a 50-pA depolarizing step when recorded at ≤10 and 12 weeks in culture. Not statistically different after two-way ANOVA. B Representative responses of active cells from control (left) and patients (right) to a 90-pA depolarizing step when recorded at ≤10 and 12 weeks in culture. The bars at the bottom represent the spike times of different cells after the pulse start (dashed line). Each row corresponds to a different cell. C, Input resistance of active cells recorded from patient- and control-derived cultures, significant main effect of disease (*p < 0.05). D, Left, representative current trace of an active cell in response to a depolarizing pulse to 25 mV from a holding potential of ‑90 mV indicating how potassium current amplitude was measured. Right, amplitude of delayed non-inactivating outward potassium currents for patient and control cells recorded at < 10 and 12 weeks in culture. Not statistically different after two-way ANOVA.. [file 13287_2021_2658_MOESM11_ESM.tiff]
