# Supplementary material for: A familiar study on self-limited childhood epilepsy patients using hIPSC-derived neurons shows a bias towards immaturity at the morphological, electrophysiological and gene expression levels
Source: Stem Cell Res Ther. 2021 Nov 25;12:590. doi: 10.1186/s13287-021-02658-2 (PMC8620942; doi:10.1186/s13287-021-02658-2)
Supplement: Supplementary file 2 — Additional file 1: Table S2. Primers used to amplify bridge regions between genes found in STEMCCA. [file 13287_2021_2658_MOESM2_ESM.docx]

| Name | Sequence | Detects | Product (pb) |
| --- | --- | --- | --- |
| *OCT4_bridge* | CAACGAGAGGATTTTGAGGC | Insertion/Silencing | 560 |
| *KLF4_bridge* | ATCGTTGAACTCCTCGGTCTCTCT |  |  |
| *SOX2_bridge* | TTGGCTCCATGGGTTCGGTG | Insertion/Silencing | 550 |
| *cMYC_bridge* | AAGGGTGTGACCGCAACGTAGG |  |  |
| Cyclophilin_F | GAAGAGTGCGATCAAGAACCCATGAC | housekeeping gene | 164 |
| Cyclophilin_R | GTCTCTCCTCCTTCTCCTCCTATCTTTACTT |  |  |
| *NAT1-U283* | ATTCTTCGTTGTCAAGCCGCCAAAGTGGAG | housekeeping gene | 223 |
| *NAT1-L476* | AGTTGTTTGCTGCGGAGTTGTCATCTCGTC |  |  |

Additional file 2: Table S2: Primers used to amplify bridge regions between genes found in STEMCCA
