# Supplementary material for: A familiar study on self-limited childhood epilepsy patients using hIPSC-derived neurons shows a bias towards immaturity at the morphological, electrophysiological and gene expression levels
Source: Stem Cell Res Ther. 2021 Nov 25;12:590. doi: 10.1186/s13287-021-02658-2 (PMC8620942; doi:10.1186/s13287-021-02658-2)
Supplement: Supplementary file 3 — Additional file 1: Table S3. Primers used to amplify endogenous pluripotency genes. [file 13287_2021_2658_MOESM3_ESM.docx]

Additional file 3: Table S3: Primers used to amplify endogenous pluripotency genes

.

| Name | Sequence | Detects | Product (pb) |
| --- | --- | --- | --- |
| OCT4_F | AACCTGGAGTTTGTGCCAGGGTTT | Pluripontency | 123 |
| *OCT4_R* | TGAACTTCACCTTCCCTCCAACCA |  |  |
| *SOX2_R* | GAGAGAGGCAAACTGGAATCAGGATCAAA | Pluripontency | 150 |
| *SOX2_F* | AGAAGAGGAGAGAGAAAGAAAGGGAGAGA |  |  |
| *NANOG_F* | CCTGAAGACGTGTGAAGATGAG | Pluripontency | 190 |
| *NANOG_R* | GCTGATTAGGCTCCAACCATA |  |  |
| *hGDF3-S243* | CTTATGCTACGTAAAGGAGCTGGG | Pluripontency | 631 |
| *hGDF3-AS850* | GTGCCAACCCAGGTCCCGGAAGTT |  |  |
| *hREX1-RT-U* | CAGATCCTAAACAGCTCGCAGAAT | Pluripontency | 306 |
| *hREX1-RT-L* | GCGTACGCAAATTAAAGTCCAGA |  |  |
| *hFGF4-RT-U* | CTACAACGCCTACGAGTCCTACA | Pluripontency | 371 |
| *hFGF4-RT-L* | GTTGCACCAGAAAAGTCAGAGTTG |  |  |
| *hpH34-S40* | ATATCCCGCCGTGGGTGAAAGTTC | Pluripontency | 243 |
| *hpH34-AS259* | ACTCAGCCATGGACTGGAGCATCC |  |  |
| *hECAT15-1-S532* | GGAGCCGCCTGCCCTGGAAAATTC | Pluripontency | 408 |
| *hECAT15-1-AS916* | TTTTTCCTGATATTCTATTCCCAT |  |  |
| *hECAT15-2-S85* | CCGTCCCCGCAATCTCCTTCCATC | Pluripontency | 606 |
| *hECAT15-2-AS667* | ATGATGCCAACATGGCTCCCGGTG |  |  |
| Cyclophilin_F | GAAGAGTGCGATCAAGAACCCATGAC | housekeeping gene | 164 |
| Cyclophilin_R | GTCTCTCCTCCTTCTCCTCCTATCTTTACTT |  |  |
|  |  |  |  |
