# Supplementary material for: A familiar study on self-limited childhood epilepsy patients using hIPSC-derived neurons shows a bias towards immaturity at the morphological, electrophysiological and gene expression levels
Source: Stem Cell Res Ther. 2021 Nov 25;12:590. doi: 10.1186/s13287-021-02658-2 (PMC8620942; doi:10.1186/s13287-021-02658-2)
Supplement: Supplementary file 4 — Additional file 1: Table S4. Primers list for EB lineage detection. [file 13287_2021_2658_MOESM4_ESM.docx]

| Primer name | Primer sequence | Lineage | Product (pb) |
| --- | --- | --- | --- |
| Alphafetoprotein_F | TGCTGGATTGTCTGCAGGATG | Endoderm | 110 |
| Alphafetoprotein_R | ACGTTCCAGCGTGGTCAGTTT |  |  |
| *CXCR4_98* | GGAGGGGATCAGTATATACACTTCAG | Endoderm | 255 |
| *CXCR4_352* | AGGAGGTCGGCCACTGACAGG |  |  |
| hBRACHYURY_S1292 | GCCCTCTCCCTCCCCTCCACGCACAG | Mesoderm | 274 |
| hBRACHYURY_AS1540 | CGGCGCCGTTGCTCACAGACCACAGG |  |  |
| *PECAM1_F* | AAGTGGAGTCCAGCCGCATATC | Mesoderm | 133 |
| *PECAM1_R* | ATGGAGCAGGACAGGTTCAGTC |  |  |
| *MAP2_F* | ACAGCAGCAGGTGGGGAATC | Ectoderm | 308/452 |
| *MAP2_R* | CGCCGAGGAGGGAGAATGG |  |  |
| *hPAX6_S1206* | ACCCATTATCCAGATGTGTTTGCCCGAG | Ectoderm | 317 |
| *hPAX6_AS1497* | ATGGTGAAGCTGGGCATAGGCGGCAG |  |  |
| Cyclophilin_F | GAAGAGTGCGATCAAGAACCCATGAC | housekeeping gene | 164 |
| Cyclophilin_R | GTCTCTCCTCCTTCTCCTCCTATCTTTACTT |  |  |
| *NAT1-U283* | ATTCTTCGTTGTCAAGCCGCCAAAGTGGAG | housekeeping gene | 223 |
| *NAT1-L476* | AGTTGTTTGCTGCGGAGTTGTCATCTCGTC |  |  |

Additional file 4: Table S4: Primers list for EB lineage detection
