# Supplementary material for: A familiar study on self-limited childhood epilepsy patients using hIPSC-derived neurons shows a bias towards immaturity at the morphological, electrophysiological and gene expression levels
Source: Stem Cell Res Ther. 2021 Nov 25;12:590. doi: 10.1186/s13287-021-02658-2 (PMC8620942; doi:10.1186/s13287-021-02658-2)
Supplement: Supplementary file 5 — Additional file 1: Table S5. Primary antibody list to label pluripotency markers in IPS lines. [file 13287_2021_2658_MOESM5_ESM.docx]

Additional file 5: Table S5: Primary antibody list to label pluripotency markers in IPS lines

| Antibody | Company | Catalog number | Dilution |
| --- | --- | --- | --- |
| Anti SOX2 | Abcam | ab130225 | 1/500 |
| Anti OCT4 | Abcam | ab105931 | 1/500 |
| Anti NANOG | Cell Signaling | 4903X | 1/500 |
| Anti TRA 1-81 | Abcam | ab16289 | 1/500 |
