# Supplementary material for: A familiar study on self-limited childhood epilepsy patients using hIPSC-derived neurons shows a bias towards immaturity at the morphological, electrophysiological and gene expression levels
Source: Stem Cell Res Ther. 2021 Nov 25;12:590. doi: 10.1186/s13287-021-02658-2 (PMC8620942; doi:10.1186/s13287-021-02658-2)
Supplement: Supplementary file 6 — Additional file 1: Table S6. Antibody list to characterize neuroepithelial tissue. [file 13287_2021_2658_MOESM6_ESM.docx]

Additional file 6: Table S6: Antibody list to characterize neuroepithelial tissue

| Antibody | Company | Catalog number | Dilution |
| --- | --- | --- | --- |
| Beta III Tubulin | Abcam | ab78078 | 1/500 |
| GFAP | Dako | Z0334 | 1/500 |
| OTX2 | Abcam | ab130238 | 1/500 |
| DCX | Abcam | ab18723 | 1/1000 |
| GAD67 | Abcam | ab26116 | 1/500 |
| PAX6 | Abcam | ab5790 | 1/500 |
| MAP2 | Sigma | M9942 | 1/500 |
| LHX2 | Abcam | ab130256 | 1/500 |
| VGLUT-1 | Abcam | ab104898 | 1/500 |
| FGD6 | Abcam | ab51217 | 1/500 |
| TAU-1 | Millipore | MAB3420 | 1/500 |
| Rhodamine phalloidin | Invitrogen | R415 | 1/1000 |
| Synapsin-1 | Abcam | ab1543 | 1/500 |
