# Supplementary material for: A familiar study on self-limited childhood epilepsy patients using hIPSC-derived neurons shows a bias towards immaturity at the morphological, electrophysiological and gene expression levels
Source: Stem Cell Res Ther. 2021 Nov 25;12:590. doi: 10.1186/s13287-021-02658-2 (PMC8620942; doi:10.1186/s13287-021-02658-2)
Supplement: Supplementary file 7 — Additional file 1: Table S7. Antibody list for EB lineage detection. [file 13287_2021_2658_MOESM7_ESM.docx]

Additional file 7: Table S7: Antibody list for EB lineage detection

| Antibody | Company | Catalog number | Lineage | Dilution |
| --- | --- | --- | --- | --- |
| Anti DCX | Abcam | ab18723 | Ectoderm | 1/500 |
| Anti BIII tubulin | Abcam | ab78078 |  | 1/500 |
| Anti Desmin | SCBT | sc-271677 | Mesoderm | 1/200 |
| Anti Troponin | Abcam | ab47003 |  | 1/500 |
| Anti AFP | Abcam | ab54745 | Endoderm | 1/500 |
| Anti GATA4 | SCBT | sc-25310 |  | 1/200 |
