# Supplementary material for: A familiar study on self-limited childhood epilepsy patients using hIPSC-derived neurons shows a bias towards immaturity at the morphological, electrophysiological and gene expression levels
Source: Stem Cell Res Ther. 2021 Nov 25;12:590. doi: 10.1186/s13287-021-02658-2 (PMC8620942; doi:10.1186/s13287-021-02658-2)
Supplement: Supplementary file 8 — Additional file 1: Table S8. Primer sequence to detect FGD6 mutation in IPS and derived cell lines. [file 13287_2021_2658_MOESM8_ESM.docx]

Additional file 8: Table S8: Primer sequence to detect FGD6 mutation in IPS and derived cell lines

| Primer | Sequence | Gene of interest |
| --- | --- | --- |
| FGD6_234F | GCAGCCGAGATAAAGAAGCC | FGD6 |
| FGD6_1185R | GTGTCTTTCTGGGCTTGGGA | FGD6 |
| FGD6_genF | GAAAACAGTAAAATTGATGAGACTTTG | FGD6 |
| FGD6_genR | GAATTCCCCGGTTCTTCAG | FGD6 |
